# Correlation between B-Type Natriuretic Peptide and Functional/Cognitive Parameters in Discharged Congestive Heart Failure Patients

**DOI:** 10.1155/2015/239136

**Published:** 2015-04-22

**Authors:** Laura Leto, Marzia Testa, Mauro Feola

**Affiliations:** ^1^School of Geriatrics, University of Medicine, 10100 Turin, Italy; ^2^Cardiovascular Rehabilitation-Heart Failure Unit, Ospedale SS Trinita, 12045 Fossano, Italy

## Abstract

The determination of B-type natriuretic peptides (BNP) may have a role in the diagnosis of heart failure (HF) or guiding HF therapy. This study investigated the role of BNP determination in a cohort of elderly patients admitted to hospital with acute decompensated HF and its correlation with main demographic, clinical, and instrumental data and evaluated possible association with major outcome such as mortality or readmission after a 6-month period of follow-up. *Methods*. From October 2011 to May 2014 consecutive patients admitted to our unit with symptoms of acute HF or worsening of chronic HF entered the study collecting functional, echocardiographic, and hydration parameters. Correlation between BNP and main parameters was analysed, as well as the mortality/6-month readmission rate. *Results*. In 951 patients (mean age 71 ys; 37% females) a positive correlation was obtained between BNP and age, creatinine levels, NYHA class at admission and discharge, and levels of hydration; an inverse, negative correlation between BNP and sodium levels, LVEF, distance performed at 6MWT at admission and at discharge, and scores at MMSE at admission and discharge emerged. BNP levels at admission and at discharge were furthermore clearly associated with mortality at 6 months (Chi-square 704.38, *p* = 0.03) and hospital readmission (Chi-square 741.57, *p* < 0.01). *Conclusion*. In an elderly HF population, BNP is related not only with clinical, laboratory, and instrumental data but also with multidimensional scales evaluating global status; higher BNP levels are linked with a worse prognosis in terms of mortality and 6-month readmission.

## 1. Introduction

B-type natriuretic peptide (BNP) is a 32-amino acid peptide primarily secreted by the cardiac ventricles in response to increased myocardial wall stress due to volume overload or higher end-diastolic pressure inside the ventricle itself. BNP has a diuretic, natriuretic, and vasodilating effect. BNP levels rise primarily in the presence of left ventricular dysfunction: the most recent guidelines suggest measuring it to support clinical decision making regarding the diagnosis of heart failure (HF), especially when diagnosis is uncertain [1]. The determination of natriuretic peptides (NP) may have a role also to guide HF therapy [1]. Both HF with reduced ejection fraction (EF) and HF with preserved EF can be associated with increased BNP levels, but also other clinical cardiac and noncardiac conditions can be linked with higher NP concentrations. Increasing levels of NP predict worse prognosis in a linear fashion across all stages of HF [2]; furthermore, decrease of NP concentrations is associated with a better prognosis than failure to decrease or increase [3]. However, no definitive data are established about the utility of a serial determination of NP levels in decreasing mortality or readmission. The present study aimed to investigate the role of BNP determination in a cohort of elderly patients admitted to hospital with acute decompensated HF and its correlation with main demographic, clinical, and instrumental data and to evaluate possible association with major outcome such as mortality or readmission after a 6-month period of follow-up.

## 2. Patients and Methods

From October 2011 to May 2014 consecutive patients admitted to our unit with symptoms of acute HF or worsening of chronic HF were asked to enter the study. HF was defined according to the presence of two major criteria or one major criterion and two minor criteria of the Framingham classification. Etiology of HF was researched and its severity according to NYHA (New York Heart Association) functional class was recorded; an echocardiogram was performed in all patients. A complete blood test was obtained; creatinine clearance according to Cockroft-Gault formula was calculated. Levels of BNP were measured at admission in all patients; 403 subjects underwent a BNP evaluation also at discharge. Body mass index (BMI) was calculated as weight in kilograms divided by the square of height in meters.

Hydration status was evaluated through a bioimpedance technique while performance was analysed with 6-minute walking test at discharge after compensation was achieved.

### 2.1. Doppler Echocardiography

Echocardiograms were performed with a Vivid 7 computed sonography system (GE Medical Systems, Waukesha, Wisconsin, USA) according to the recommendations of the American Society of Echocardiography. Two-dimensional apical 2- and 4-chamber views were used for volume measurements; left ventricular ejection fraction (LVEF) was calculated with modified Simpson's method using biplane apical (2- and 4-chamber) views. The LV end-diastolic volume and the LV end-systolic volume were recorded. All the echo examinations were performed by expert operators blinded to the results of BNP assay; the intraobserver variability in the evaluation of LVEF was found to be <5%. Echocardiographic measurements including LV end-diastolic diameter and the diastolic thickness of the ventricular septum and the posterior LV wall were determined according to the American Society Echocardiography recommendations [4]. Systolic dysfunction was defined as a level of LVEF <50%. The definition of restrictive filling pattern (grade 3) was a predefined modification of classifications used in prior studies:  *E*/*A* ≥ 2, DT ≤ 150 msec, *S*/*D* ratio < 1, and AR > 35 cm/sec. All these criteria should be verified to define the restrictive filling pattern. The other diastolic filling patterns were classified as follows: grade 1 (abnormal relaxation) when *E*/*A* < 1 with DT > 240 ms; grade 2 (pseudonormal) when *E*/*A* is between 0.75 and 1.5, DT is between 160 and 240 ms, and finally *E*/*Ea* > 15 [5]. The presence of this diastolic pattern with LVEF ≥50% was defined as an isolated diastolic dysfunction.

### 2.2. BNP Assay

All blood samples were collected by venopuncture and immediately analysed with the bedside Triage B-type natriuretic fluorescence immunoassay (Biosite Diagnostics, La Jolla, CA, USA). The Triage Meter is used to measure BNP concentration by detecting a fluorescent emission that reproduces the amount of BNP in the blood. Two hundred and fifty *μ*L of whole blood was added to the disposable device; then the cells were filtered and separated from the plasma with BNP, which entered a reaction chamber, containing fluorescent BNP antibodies. After 2-min incubation, the BNP-antibody mixture migrated to an area containing immobilised antibodies and remained fixed. The unbound fluorescent antibodies were washed away by the excess sample fluid. Then, the Triage Meter measured the fluorescent intensity of the BNP assay area. The assay results were complete in 15 minutes.

### 2.3. Six-Minute Walk Test

The 6MWT was performed in the discharging day according to the ATS Statement of the American Thoracic Society [6]. CHF patients able to walk underwent 6MWT if they did not meet the exclusion criteria (unstable angina and myocardial infarction during the previous month, resting heart rate >120, systolic blood pressure >180 mmHg, or diastolic blood pressure >100 mmHg).

### 2.4. Bioelectrical Impedance Vectorial Analysis (BIVA)

Assessment of body fluid status was carried out using an electrical impedance analyser and BodyGram 2.1 software (Akern Pontassieve, Florence, Italy). The bioelectrical parameters of resistance, reactance, and phase angle were obtained with an electric alternating current flux of 800 microA and an operating frequency of 50 kHz. Whole body impedance measurements were taken by using a standard position of outer and inner electrodes on the right hand and foot. The entire procedure was performed according to the indications of the National Institutes of Health Technology assessment conference statements [7]. Bioelectrical impedance evaluates some basic properties of the body by measuring resistance, reactance (a form of opposition that electronic components exhibit to the passage of alternating current counterpart of direct current and indicates an absolute amount of body cell mass). Bioelectrical impedance is normally used to estimate the volumes of body fluid compartments allowing determining total body water and the ratio between extracellular and total body water. Resistance and reactance were always corrected for the patients' height. The clinical support of this method has been implemented using the vector analysis (Bioelectrical Impedance Vector Analysis, BIAVECTOR) [8]. The backward or forward position of parallel vectors to the major axis of ellipses is normally correlated with dehydration or hyperhydration. The normal value of hydration has been set at value 73.3% and the area within +1 SD was considered a satisfactory criterion for discharging CHF patients.

Functional status was evaluated through validated tools as activities of daily living (ADL) index [9], developed by Katz, which explores the level of dependence or independence in six basic tasks, that is, bathing, toileting, dressing, continence, movement, and feeding, Barthel index [10] exploring ADL functions and mobility, and instrumental activities of daily living (IADL) index [11] that involves more complex abilities such as use of phone, doing shopping, use of transports, and drugs management.

All patients underwent a complete examination of cognitive status with Mini Mental State Examination (MMSE) [12] while the presence of mood disorders was explored with Geriatric Depression Scale (GDS) [13]. The MMSE is an easy test that takes approximately 10 minutes to be administered and that covers seven different cognitive domains: temporal and spatial orientation, immediate and delayed memory, attention, calculation, and language (denomination, repetition, comprehension, reading, writing, and constructional apraxia). The maximum score is 30 and scores of 25–30 are considered normal; total scores from 21 to 24 indicate mild impairment, from 10 to 20 moderate impairment, and <10 severe impairment. A correction can be applied according to age and years of schooling. The GDS is a 15-item self-report measure of depression in older adults; the participants are asked to answer “yes” or “no” according to how they felt in the past week. No questions on semantic expression of depressed mood are involved. Ten questions indicate the presence of depression when answered positively, while the rest indicate depression when answered negatively; one point is assigned to each answer if the presence of depression is suggested. Scores of 0–4 are considered normal, 5–8 indicate mild depression, 9–11 indicate moderate depression, and 12–15 indicate severe depression. It takes approximately 5–7 minutes to be administered and its simplicity and rapid application enable the scale to be used with ill or moderately cognitively impaired individuals. The Minnesota Living with Heart Failure Questionnaire (MLWHFQ) [14] was used to assess quality of life in this population: it measures the effects of symptoms, functional limitations, and psychological distress on an individual quality of life. It consists of questions that assess the impact of frequent physical symptoms, the effects of HF on physical/social functions, and side effects of treatments, hospital stays, and costs of care.

### 2.5. Clinical Follow-Up

Death by any cause, cardiac transplantation, and worsening heart failure requiring readmission to the hospital were considered cardiovascular events. Data regarding the occurrence of cardiovascular events were collected from multiple sources in all patients.

### 2.6. Statistical Analysis

Counts and percentages for categorical and ordinal variables were calculated while continuous variables were expressed as mean ± standard deviation (SD). BNP levels were evaluated both as a continuous variable and as a categorical variable (based on cut-off values). According to the plasma BNP value 2 groups were created: <300 pg/mL and ≥300 pg/mL in order to evaluate the role of different levels of plasma BNP on clinical, laboratory, and instrumental parameters and the occurrence of cardiac events in the follow-up. Pearson's correlation coefficient was calculated to describe the linear correlation between BNP levels and multiple parameters. The comparison between different means in the two BNP groups was verified through the independent two-sample *t*-test (Student's *t*-test). Pearson's Chi-square test was used to evaluate the association between BNP levels and categorical outcome variables (mortality, hospital readmission). A normal distribution was assumed. A *p* value <0.05 was considered significant. Analyses were performed using SPSS software per Windows, release 22.0, SPSS Inc., Chicago, USA.

## 3. Results

Data on 951 patients were recorded in the stated period.  Table 1 shows main demographic, clinical, and instrumental data of the sample. Mean age was 70.8 years, and 74.2% of the sample was older than 65 years while 36.8% was older than 75. More than one-third of the sample was female. In 46.8% the etiology of heart failure was ischaemic, 96 subjects underwent previous percutaneous angioplasty, and 251 underwent by-pass graft. An automatic implantable cardioverter defibrillator (AICD) was implanted in 8.6% of the sample. In more than half of the examined population a LVEF lower than 50% emerged. Functional status was minimally impaired with a mean score at ADL index of 5.4 lost functions and a total Barthel score of 86.3. Patients in the sample presented a minimally compromised cognitive function (total score at MMSE of 27.3) and a depressed mood (total score at GDS of 6.4 on a 15-item scale). In the 6-month period of follow-up, 76 patients died while 57 (6% of the sample) underwent a new hospital readmission. Mean BNP level at admission was 549.1 pg/mL, and at discharge it was 408.9 pg/mL; in 403 patients BNP levels were measured both at admission and at discharge: in 326 (80.9%) they were lower at discharge while in 77 subjects no difference or even higher levels were present at discharge.

At univariate analysis, a positive correlation was obtained between BNP and age (*r* = 0.15; *p* < 0.01), creatinine levels (*r* = 0.29; *p* < 0.01), NYHA class at admission and discharge (*r* = 0.36; *p* < 0.01; *r* = 0.21; *p* < 0.01), and levels of hydration at bioimpedance analysis (*r* = 0.37; *p* < 0.01); an inverse, negative correlation between BNP and sodium levels (*r* = −0.15; *p* < 0.01), LVEF (*r* = −0.46; *p* < 0.01), scores at ADL index (*r* = −0.20; *p* < 0.01) and Barthel index (*r* = −0.18; *p* < 0.01), distance performed at 6MWT at admission and at discharge (*r* = −0.25; *p* < 0.01 and *r* = −0.30; *p* < 0.01), and scores at MMSE at admission and discharge (*r* = −0.17; *p* = 0.02) emerged. Patients with BNP level higher than 300 pg/mL were older, with a worse renal function, a lower haemoglobin count, lower LVEF, a worse functional status measured with Barthel index, a higher hydration level assessed with bioimpedance analysis, a worse physical (at 6MWT) and cognitive (at MMSE) performance, and a depressed mood (at GDS), and experienced longer hospital stay (Table 2).

BNP levels were significantly lower in patients with HF with preserved LVEF compared to patients with HF with reduced LVEF (303.1 pg/mL versus 839.1 pg/mL, *p* < 0.01). BMI was calculated in 90 subjects: mean value was 25.5 and 36 patients (40%) were overweight while 13.3% of the sample was obese. Overweight (BMI > 25) and obese (BMI > 30) patients have lower BNP levels even if no statistical significance was reached, also because of the small sample size. In patients with diabetes, lower BNP concentrations were recorded (1049 pg/mL versus 1414 pg/mL) even if no statistical significance emerged (Table 3).

BNP levels at admission and at discharge were furthermore clearly associated with mortality at 6 months (Chi-square 704.38, *p* = 0.03) and hospital readmission (Chi-square 741.57, *p* < 0.01).

## 4. Discussion

BNP is secreted by the cardiac ventricles in response to increased myocardial wall stress due to volume overload or higher end-diastolic pressure inside the ventricle itself. The activation of BNP gene in cardiomyocytes produces a precursor propeptide (proBNP_108_) further cleaved into the biologically inert amino terminal fragment (NT-proBNP) and the biologically active BNP. BNP has a diuretic and natriuretic effect increasing the glomerular filtration rate and decreasing sodium reabsorption in the collecting duct thus improving sodium excretion (downregulation of the rennin-angiotensin-aldosterone system); furthermore it decreases peripheral vascular resistance and increases smooth cells relaxation thus resulting in a vasodilating effect. In our sample, sodium levels were lower in subjects with higher BNP concentration even if no statistical significance was reached (data not shown).

NT-proBNP and BNP differ also for half-life (120 versus 20 minutes) thus resulting in higher levels of circulating NT-proBNP; they are both cleared by the kidney [15]. BNP levels rise primarily in the presence of left ventricular dysfunction: the most recent clinical guidelines on heart failure (HF) management suggest its measurement to support clinical decision regarding the diagnosis and to establish prognosis or disease severity in ambulatory patients with dyspnea [16], in chronic HF [17], in hospitalized subjects [18, 19], and in acute decompensated HF (ADHF) [20] (Class I, Level of Evidence A). According to ACCF/AHA guidelines [1], natriuretic peptide- (NP-) guided HF therapy can be useful to achieve optimal dosing of guideline-directed medical therapy in euvolemic patients followed in a structured disease management program [21–24] (Class IIa, Level of Evidence B) but it is not well established for ADHF [25] (Class IIb, Level of Evidence C). Furthermore, the usefulness of serial measurement of BNP or NT-proBNP to reduce hospitalization or mortality in patients with HF is not well established [11, 12, 26] (Class IIb, Level of Evidence B). Both systolic dysfunction and HF with preserved ejection fraction (HFpEF) may be linked to elevated BNP and NT-proBNP levels, although HFpEF may be associated with lower concentrations of both peptides. Also in our sample of elderly hospitalized patients with ADHF, BNP levels were significantly lower in patients with HFpEF compared to patients with HF with reduced LVEF. However, also other conditions are associated with higher BNP levels: cardiac disease such as acute coronary syndrome, valvular heart disease [27], pericardial disease, atrial fibrillation [28], myocarditis, cardiac surgery and noncardiac ones such as renal failure [29], liver cirrhosis with ascites, pulmonary hypertension, and severe pneumonia. Advancing age may be linked with higher concentrations also without overt HF [30]; on the contrary obesity may result in lower BNP or NT-proBNP serum levels even when HF is present [31, 32]. In our sample BNP levels were positively associated with age; furthermore, overweight and obese patients had lower BNP concentrations if compared to subjects with BMI <25.

In our sample of ADHF patients, BNP was related to functional, cognitive, and behavioral scales that are part of a multidimensional approach usually applied in elderly comorbid patients in order to assess global status. BNP has consistent data as regards the relationship with cognitive impairment in general elderly population [33, 34], in individuals with cardiovascular disease [35], and in patients with HF [36, 37]. In a cohort of 464 individuals, mean age of 79 years, MMSE was administered at baseline and after a follow-up period of 5 years: BNP was the only variable connected with decline of MMSE over time and it was associated with new diagnosis of dementia, defined according to DSM-IV criteria and to guidelines with an OR = 1.53 (95% CI 1.09–2.16, *p* = 0.013) [33]. In the Rancho Bernardo Study, a population-based study of the epidemiology of chronic disease on 950 older individuals with an average age of 77 years, elevated NT-proBNP values were independently associated with poor cognitive function on MMSE (OR 2.0, 95% CI 1.1–3.6, and *p* = 0.02) [34]. Regarding individuals with HF, in sixty patients hospitalized for exacerbation of HF (mean age 65.5 years, mean LVEF 32.9, and average BNP plasma level 683.3 pg/mL) BNP was related with MMSE (*r* = 0.12, *p* = 0.02) but not with other cognitive tests [35–37]. In our sample of ADHF patients with an almost normal mean MMSE score, BNP concentration was related with total MMSE score with subjects with higher NP levels experiencing more cognitive decline.

As for prognosis, increasing levels of NP were showed to predict worse prognosis in a linear fashion across all stages of HF [2]. Determination of NP plasmatic levels may be important both as an initial evaluation and during follow-up: according to observational studies a 30% decrease of NP levels is associated with a better prognosis than failure to decrease or increase [3]. In our sample BNP levels at admission and at discharge were related to a worse prognosis as for mortality and readmission at 6-month follow-up.

## 5. Conclusion

In conclusion, in an elderly HF population, hospitalized with signs and symptoms of congestive HF (more than a third of the sample females, with a moderate neurohormonal activation and more than half of the sample presenting an impaired LVEF), BNP is related not only with clinical, laboratory, and instrumental data that are usually assessed in HF patients but also with multidimensional scales evaluating global status; furthermore, also in this large single-centre population, higher BNP levels are linked with a worse prognosis in terms of mortality and readmission at 6 months.

## Figures and Tables

**Table 1:** Main demographic, clinical, and laboratory data.

Clinical features	Patients (*n* = 951)	Range
Age (years)	70.8 ± 10.3	30–92
Female	352 (37%)	
Mean ejection fraction (%) (i) Ejection fraction ≤50%	46.9 ± 14.4 548 (57.6%)	10–89
Heart failure severity (NYHA class at admission)		
(i) NYHA II	388 (40.8%)	
(ii) NYHA III	326 (34.3%)	
(iii) NYHA IV	90 (9.5%)	
BNP level at admission (pg/mL)	549.1 ± 898.2	4–17860
Creatinine plasma level (mg/dL)	1.17 ± 0.6	0.4–6.3
Creatinine clearance (according to Cockroft-Gault equation) (mL/min)	62.3 ± 25.4	15–129
Sodium serum level (mg/dL)	139.6 ± 7.3	120–152
Body mass index (BMI) (*n* = 90)	25.5 ± 5.4	15–39
Heart failure etiology		
(i) Coronary artery disease	445 (46.8%)	
(a) Previous by-pass graft surgery	251 (26.4%)	
(b) Previous percutaneous revascularization	96 (10.1%)	
(ii) Valvular cardiomyopathy	258 (43.7%)	
Atrial fibrillation	199 (21%)	
AICD	82 (8.6%)	
6-minute walking test (meters)	310.7 ± 93.0	50–570
Barthel index	86.3 ± 23.4	0–100
Score on MMSE	27.3 ± 3.2	10–30
GDS 15 items	6.4 ± 3.5	0–15

NYHA: New York Heart Association; BNP: B-type natriuretic peptide; AICD: automatic implantable cardioverter defibrillator; MMSE: Mini Mental State Examination; GDS: Geriatric Depression Scale.

**Table 2:** Differences in clinical, laboratory, and instrumental parameters according to BNP levels.

	BNP ≥300 pg/mL	BNP <300 pg/mL	*p*
Age (years)	72.4 ± 9.6	69.3 ± 10.7	<0.001
Creatinine level (mg/dL)	1.3 ± 0.7	1.0 ± 0.4	<0.001
Creatinine clearance (mL/min)	55.7 ± 23.7	70.2 ± 25.3	<0.001
Haemoglobin (g/dL)	11.4 ± 1.8	12.2 ± 2.0	0.001
LVEF (%)	40.7 ± 14.7	53.1 ± 11.2	<0.001
Barthel index at admission (score)	81.2 ± 26.0	91.1 ± 19.7	<0.001
Hydration level at BIVA at admission (%)	77 ± 5.3	73.9 ± 2.4	<0.001
6MWT at admission (meters)	297.6 ± 90.1	321.8 ± 94.2	0.015
MMSE (score)	26.7 ± 3.6	28.0 ± 2.5	<0.001
GDS (score)	8.2 ± 4.7	6.9 ± 3.3	0.046
Length of hospital stay (days)	13.3 ± 8.1	11.1 ± 7.2	<0.001

LVEF: left ventricular ejection fraction; BIVA: bioelectrical impedance vectorial analysis; 6MWT: 6-minute walking test; MMSE: Mini Mental State Examination; GDS: Geriatric Depression Scale.

**Table 3:** Differences in BNP levels in overweight or obese and normal weight patients and in patients with or without diagnosis of diabetes.

	BNP levels (pg/mL)	*p*
Overweight and obese (BMI ≥ 25)	570.4 ± 678.3	0.13
Normal weight (BMI < 25)	1288.1 ± 878.7	

Presence of diabetes	1049.2 ± 1240.1	0.53
Absence of diabetes	1414.1 ± 1875.1	

BNP: B-type natriuretic peptide; BMI: body mass index.
